# SPEECH-LANGUAGE PATHOLOGISTS’ PRACTICE PATTERNS IN THE MANAGEMENT OF COGNITIVE IMPAIRMENT IN PARKINSON’S DISEASE

**DOI:** 10.1093/geroni/igad104.2630

**Published:** 2023-12-21

**Authors:** Vanessa Neal, Nan Musson

**Affiliations:** Department of Veteran Affairs, Orlando, Florida, United States; Department of Veteran Affairs, Washington, District of Columbia, United States

## Abstract

Cognitive impairment is common in patients with Parkinson’s disease (PD) and can have significant impact on quality of life. Speech-language pathologists (SLPs) are qualified to treat cognitive impairment in PD, yet previous studies have shown SLPs tend to place greater focus on other impairment areas (e.g., dysarthria) and may lack confidence treating cognition. This study sought to better understand practice patterns in PD cognitive treatment among Veteran Affairs SLPs (N=14) participating in a national quality improvement project. Using a mixed-methods design, we examined SLPs’ use of formal assessments (e.g., standardized screening) and patient-reported outcome measures for cognition. We also examined SLPs’ clinical recommendations and perspectives toward providing cognitive services. Data was obtained using REDCap and semi-structured interviews. Results showed formal cognitive assessments were completed on 45% of patients (N=191) and patient-reported outcome measures were completed with 36% of patients. Of the patients formally assessed (n=86), 55 scored as having some degree of cognitive impairment and 17 were ultimately enrolled in cognitive treatment. The most common reasons SLPs provided for not treating cognition included: patient declining treatment, supportive home environment, needing further testing, or other medical needs (e.g., sleep management). Overall, results suggest cognitive management was a low priority among clinicians and patients. SLPs reported barriers to providing cognitive treatment included patient desire to participate and therapeutic time constraints when also providing speech and/or swallowing services. Further research is needed to increase integration of cognitive services into SLP treatment of PD and identify strategies to increase patient buy-in of cognitive treatment.

